# Influence of Artifact Removal on Rare Species Recovery in Natural Complex Communities Using High-Throughput Sequencing

**DOI:** 10.1371/journal.pone.0096928

**Published:** 2014-05-06

**Authors:** Aibin Zhan, Wei Xiong, Song He, Hugh J. MacIsaac

**Affiliations:** 1 Research Center for Eco-Environmental Sciences, Chinese Academy of Sciences, Beijing, China; 2 Great Lakes Institute for Environmental Research, University of Windsor, Windsor, Ontario, Canada; Zhejiang University, China

## Abstract

Large-scale high-throughput sequencing techniques are rapidly becoming popular methods to profile complex communities and have generated deep insights into community biodiversity. However, several technical problems, especially sequencing artifacts such as nucleotide calling errors, could artificially inflate biodiversity estimates. Sequence filtering for artifact removal is a conventional method for deleting error-prone sequences from high-throughput sequencing data. As rare species represented by low-abundance sequences in datasets may be sensitive to artifact removal process, the influence of artifact removal on rare species recovery has not been well evaluated in natural complex communities. Here we employed both internal (reliable operational taxonomic units selected from communities themselves) and external (indicator species spiked into communities) references to evaluate the influence of artifact removal on rare species recovery using 454 pyrosequencing of complex plankton communities collected from both freshwater and marine habitats. Multiple analyses revealed three clear patterns: 1) rare species were eliminated during sequence filtering process at all tested filtering stringencies, 2) more rare taxa were eliminated as filtering stringencies increased, and 3) elimination of rare species intensified as biomass of a species in a community was reduced. Our results suggest that cautions be applied when processing high-throughput sequencing data, especially for rare taxa detection for conservation of species at risk and for rapid response programs targeting non-indigenous species. Establishment of both internal and external references proposed here provides a practical strategy to evaluate artifact removal process.

## Introduction

Significant human-mediated global changes are driving rates of species extinction that greatly outpace background rates in the fossil record [1]–[2]. Evidence is building that the mass extinction of species which is currently underway alters key processes important to productivity and sustainability of ecosystems [3]–[5]. For biodiversity conservation and management purposes, there exists an urgent need to understand causes and consequences of large-scale biodiversity changes [2], [4], [6].

Biodiversity conservation and management are seriously challenged by gaps in taxonomic coverage of existing biodiversity information, or heterogeneity in geographical and/or habitat coverage [7]. Thus far, less than 2% biodiversity on the Earth has been described (i.e. <2 million out of >100 million estimated species) [8]–[9]. In addition, larger organisms and terrestrial species are usually identified and described first, mainly due to easy sampling and/or identification. Such a bias leaves a huge knowledge gap of biodiversity in aquatic communities, especially for small organisms [10]–[11]. However, conservation plans must accurately assess community composition and structure to know what species are being threatened and what non-indigenous species have been introduced into local environments, as well as which conservation plans are likely to be most effective to protect threatened species and to eradicate invading non-indigenous species [6], [11]–[12]. One of the major technical challenges for developing effective conservation plans is identification of species with small population size and/or small organisms in habitats such as plankton in aquatic ecosystems [11]–[12].

The advent of massively parallel high-throughput sequencing technologies such as 454 pyrosequencing has revolutionized biodiversity assessment in complex communities, notably in those dominated by small organisms, with some assessments reporting orders of magnitude more biodiversity than was previously recognized [10]–[11], [13]. However, several technical problems that could lead to artificial inflation of biodiversity estimates have been identified for these technologies [14]. For example, sequencing artifacts such as nucleotide base calling errors in large datasets could greatly inflate biodiversity estimates [15]–[16]. In order to eliminate overestimation caused by sequencing artifacts, high-throughput sequencing data is usually subjected to stringent read quality filtering [15]–[20], and several studies have suggested filtering thresholds for further data processing. For example, Kunin *et al.* [16] conducted deep pyrosequencing of a single species (*Escherichia coli* MG1655) and suggested that a 0.2% error probability (i.e. *Q* = 27) and a clustering threshold of 97% identity be applied when grouping Operational Taxonomic Units (OTUs) for community profiling. A more recent study suggested even higher score-based filtering stringencies, such as *Q*>30 for the hypervariable V4 region of the nuclear small subunit ribosomal DNA [19]. However, many issues related to the artifact removal process, such as the influence of artifact removal on rare species recovery, have not been evaluated in natural complex communities owing to many technical/computation challenges, numerous undescribed species and lack of reliable references in natural communities. Rare taxa represented by low-abundance sequences are expected to be eliminated first during the sequence filtering process, mainly due to the low number of sequences in final datasets.

In this study, we employed both internal and external references to evaluate the influence of artifact removal on rare species recovery for 454 pyrosequencing data derived from complex plankton communities collected from both freshwater and marine habitats. For the internal reference, we chose Operational Taxonomic Units (OTUs) with high similarities (similarity ≥99% and query coverage ≥99%) to available species in GenBank. At such a high level of similarity and coverage, the chosen OTUs may represent real taxa in communities, rather than PCR- and/or sequencing-mediated artifacts, making them reliable as internal references. For the external reference, we spiked known indicator species into complex plankton communities using concentration gradients. For both methods, we used a series of filtering stringencies to examine whether these references could be recovered as filtering stringencies increased. We aim to evaluate the influence of artifact removal on rare species recovery based on high-throughput sequencing techniques.

## Materials and Methods

### Ethics Statement

Plankton samples were collected from one marine harbour: Bayside in Nova Scotia on the Atlantic coast of Canada (45°7′–45°10′N, 67°7′–67°9′W), and one freshwater harbour: Nanticoke in Ontario on Great Lakes (42°47′–42°48′N, 80°2′–80°3′W). No specific permits were required for the described field sampling. Sampling sites did not cover protected or private lands. The field studies did not involve endangered and/or protected species.

### Field Sampling

We used six geo-referenced, 80-µm oblique plankton nets to tow from the bottom to the water surface for both harbours. The collected plankton samples were immediately homogenized into a single sample, preserved in 100% ethanol, and stored at −20°C prior to genetic analyses.

### External Reference Setup

**Figure 1:**
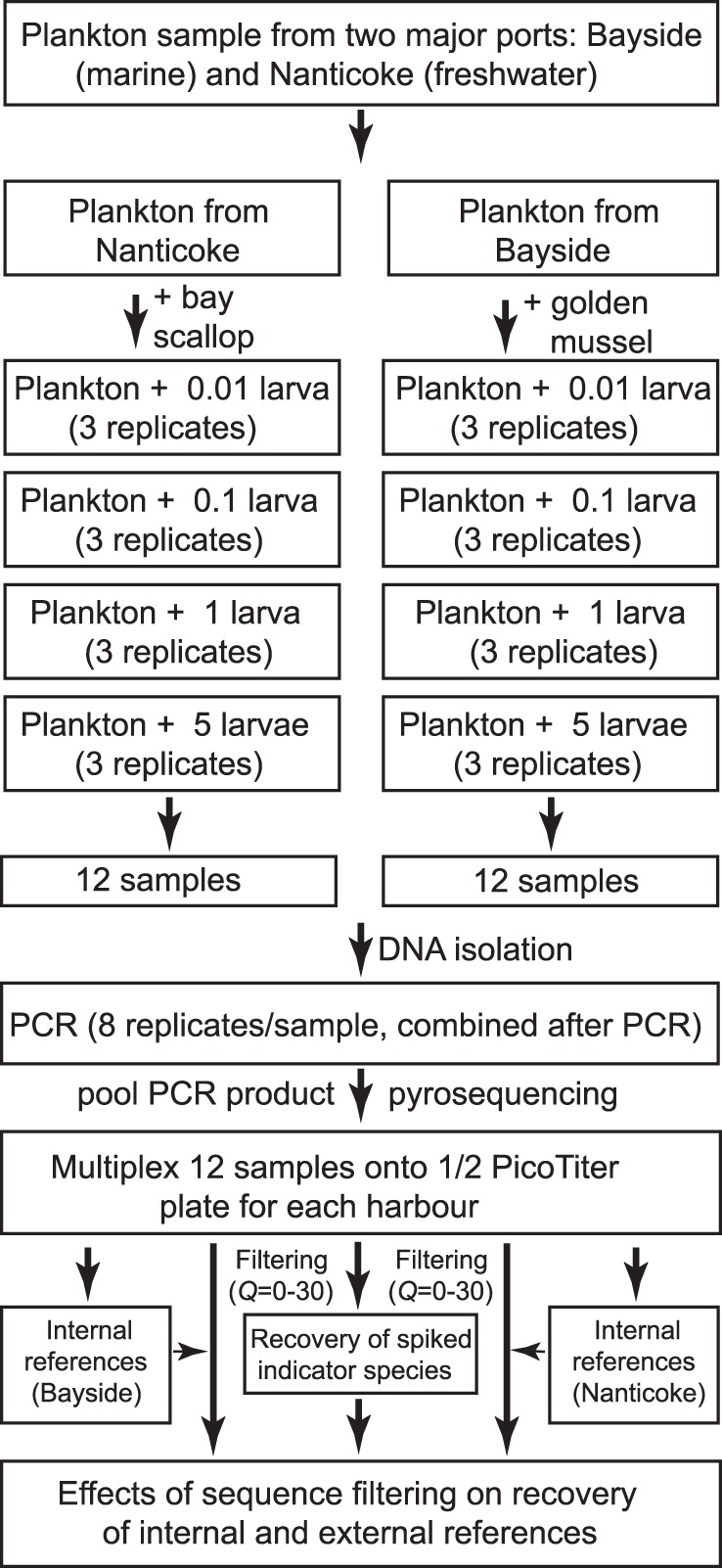
Methodological flow chart for setting up internal and external references for evaluating the influence of artifact removal on rare species detection in complex plankton communities using 454 pyrosequencing.

To set up external references, we spiked known indicator species into natural plankton communities (see reference 11 for more detail). To avoid possible errors and confusion derived from spiked species, we spiked marine species into freshwater plankton samples and freshwater species into marine plankton samples. Specifically, we spiked larvae of a freshwater mussel (golden mussel *Limnoperna fortunei*) into the marine plankton community sampled from Bayside Harbour, while larvae of a marine scallop (bay scallop *Argopecten irradians*) were spiked into the freshwater sample collected from Nanticoke Harbour (Figure 1). None of the indicator species have ever been reported in the plankton communities into which they were spiked. Larvae of the bay scallop were artificially cultured in the laboratory [21], while larvae of golden mussel were collected from the wild in South America [11]. For each spiked species, we ran three replicates and four gradients (Figure 1). All assembling procedures were performed before DNA extraction. For the gradients using >1 larva, we spiked larvae directly into plankton samples, while for those <1, we lysed one larva using 200 µL DNA lysis buffer and then added different amounts of lysed larva solution into corresponding lysed plankton samples based on serial dilution gradients. In total, we prepared 12 samples (three replicates×four gradients) for each indicator species for each harbour (Figure 1).

### DNA Isolation, PCR and Pyrosequencing

We extracted total genomic DNA using DNeasy Blood and Tissue Kit (Qiagen Inc., ON, Canada). The quality and quantity of each DNA sample were measured by a NanoDrop spectrophotometer (NanoDrop Technologies, DE, USA). For PCR, we used the primer pair Uni18S- Uni18SR for the hypervariable V4 region of the nuclear small subunit ribosomal DNA (SSU rDNA), which was specifically designed for pyrosequencing for plankton samples [11], [22]. PCRs were performed in 25 µL cocktail in eight duplicates for each sample to avoid biased amplification. Each duplicate contained 100 ng DNA, 1×PCR buffer, 2 mM Mg^2+^, 0.2 mM dNTPs, 0.4 µM each primer, and 2 U *Taq* DNA polymerase (Genscript). PCR cycling parameters consisted of an initial denaturation step at 95°C for 5 min, followed by 25 amplification cycles of 95°C for 30 s, 50°C for 30 s, 72°C for 90 s, and a final elongation step at 72°C for 10 min. We pooled and subsequently purified PCR products of duplicates using the Solid Phase Reversible Immobilization (SPRI) paramagnetic bead-based method (Agencourt Bioscience Corporation, MA, USA).

After purification, we pooled PCR products derived from 12 artificially assembled samples to form 1/2 PicoTiter plate for each harbour. To ensure approximately equal contributions from each sample, equimolar PCR products from each sample were pooled together. Samples were differentiated by a unique eight-nucleotide tag for each sample at the 5′-end of the forward primer [23]. Pyrosequencing was performed using 454 FLX Adaptor A on a GS-FLX Titanium platform (454 Life Sciences, CT, USA) by Engencore at the University of South Carolina.

### Data Analysis

Raw sequences reads were filtered using the methods implemented in pipelines Mothur [24] and UPARSE [18]. In general, we deleted low-quality sequences that: (i) did not match the tags and forward primer; (ii) contained any undetermined nucleotide (N’s); (iii) were too short (i.e. <150 bp); or (iv) contained homopolymers larger than eight. The length of each sequence read was set as 300 bp following the method in UPARSE [18]. Subsequently, nucleotides were examined one by one along their sequence reads to examine nucleotide quality, and sequences were truncated at the end of the last nucleotide before the quality score fell below the set threshold (i.e. filtering stringency), even if downstream nucleotide would again rise above the set threshold. The filtering stringencies were set from *Q* (Phred score)  = 0 to 30.

Filtered sequences were clustered into Operational Taxonomic Units (OTUs) at a commonly used cut-off value (97%) using a novel algorithm that performs chimera filtering and OTU clustering simultaneously implemented in UPARSE [18]. To set up internal references, all OTUs generated without filtering (i.e. *Q* = 0) were subjected for BLASTn searches against available database in GenBank. OTUs with minimum query coverage ≥99% and similarities ≥99% to available species in GenBank were selected as internal references. In order to assess the effects of sequence filtering on different abundance of OTUs (i.e. OTUs with different number of sequences), we divided all reference OTUs into four groups: OTUs with the number of sequences of > 100, OTUs with the number of sequences of 11∼99, OTUs with the number of sequences of 4∼10, and OTUs with the number of sequences of 1∼3 (i.e. singletons, doubletons and tripletons). For external references, after sequence reads were subjected to a series of filtering stringencies, the known spiked rare species were identified from each dilution gradient and replicate using local BLAST.

## Results

### Internal Reference

**Figure 2:**
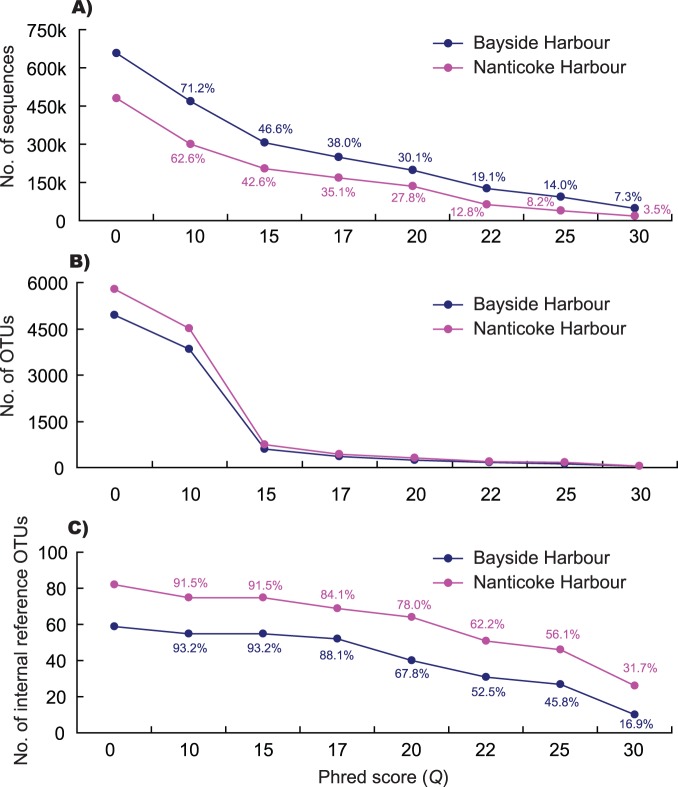
Number of sequences (A), number of Operational Taxonomic Units (OTUs, B), and number of internal reference OTUs (C) retained at a range of filtering stringencies of *Q* (Phred score)  = 0–30 for 454 pyrosequencing of two complex plankton communities collected from Bayside (marine) and Nanticoke (freshwater) Harbours. *Q* = 0 indicates that data was not filtered.

After a run of 1/2 PicoTiter plate for each harbour, a total of 656,488 and 480,962 sequences were obtained for Bayside (GenBank SRA accession: SRP036156) and Naticoke (GenBank SRA accession: SRP036187), respectively. In order to set up internal references, we grouped sequences into OTUs without filtering. We detected a large number of OTUs for both harbours: 4936 for Bayside and 5773 for Nanticoke (Figure 2B). After BLASTn, 59 and 82 OTUs derived from Bayside and Nanticoke were chosen as internal references based on the strict selection criteria (i.e. coverage ≥99% and similarity ≥99%; Table S1).

The raw sequences from both harbours were subjected to filtering at a series of stringencies (Figure 2A). As expected, the number of sequences decreased as filtering stringencies increased. In general, the percentage of sequences passing the set filtering stringencies was slightly different for the two harbours (Figure 2A). After sequences were grouped into OTUs, similarly to the pattern for sequences, the number of OTUs decreased as the stringencies increased. A sharp decrease was detected at low filtering stringencies of *Q* = 15 (Figure 2B), suggesting that sequencing artifacts can largely inflate the number of species in complex communities (i.e. α-diversity).

When sequences from both harbours were filtered with internal references, these reference OTUs were eliminated as filtering stringencies increased. Reference elimination occurred at all filtering stringencies examined, even at low *Q* values such as *Q* = 10 (Figure 2C). Moreover, more OTUs were eliminated as filtering stringencies increased. For example, 32.2% and 22.0% of reference OTUs were deleted at *Q* = 20 for Bayside and Nanticoke Harbours, respectively, while a much larger percentage of reference OTUs, i.e. 83.1% and 68.2%, was discarded at *Q* = 30 for both harbours (Figure 2C). Many of these eliminated reference OTUs had 100% similarity to species records in GenBank (Tables S1 & S2). In addition, similar to the pattern for sequences, a slight difference in elimination of reference OTUs was detected between these two harbours. For example, when compared to Nanticoke Harbour, a lower number of reference OTUs were eliminated before *Q*<20, but more after *Q*>20 for Bayside Harbour (Figure 2C).

**Figure 3:**
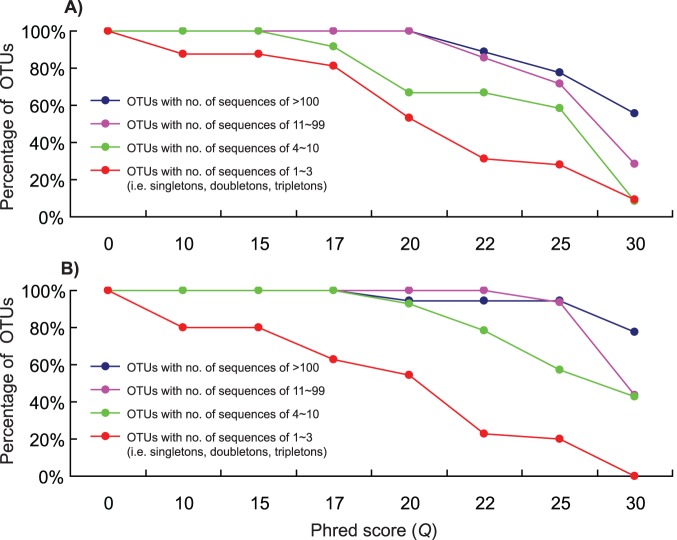
Number of internal reference Operational Taxonomic Units (OTUs) retained in four groups divided based on OTU abundance at a range of filtering stringencies of *Q* = 0–30 for 454 pyrosequencing of two complex plankton communities collected from Bayside (marine, A) and Nanticoke (freshwater, B) Harbours.

When we divided all reference OTUs into four groups based on their abundance, we found that filtering process had more influence on low abundance OTUs (i.e. OTUs with less number of sequences) for both harbours (Figure 3). Low-abundance OTUs decreased more sharply than high-abundance OTUs as filtering stringencies increased. For example, 46.9% of singletons, doubletons and tripletons were discarded at *Q* = 20; however, all OTUs with the number of sequences>10 were recovered in Bayside Harbour (Figure 3). Similarly, all singletons, doubletons and tripletons were eliminated at *Q* = 30 for Nanticoke, while more than 40% of reference OTUs were retained for the other three groups (Figure 3). When comparing the two communities, a slight difference was observed as filtering stringencies increased. For example, more than 90% of reference OTUs with the number of sequences of 4∼10 passed the quality filtering at *Q* = 20 for Nanticoke; however, a much lower ratio (66.7%) was detected for Bayside (Figure 3).

### External Reference

**Figure 4:**
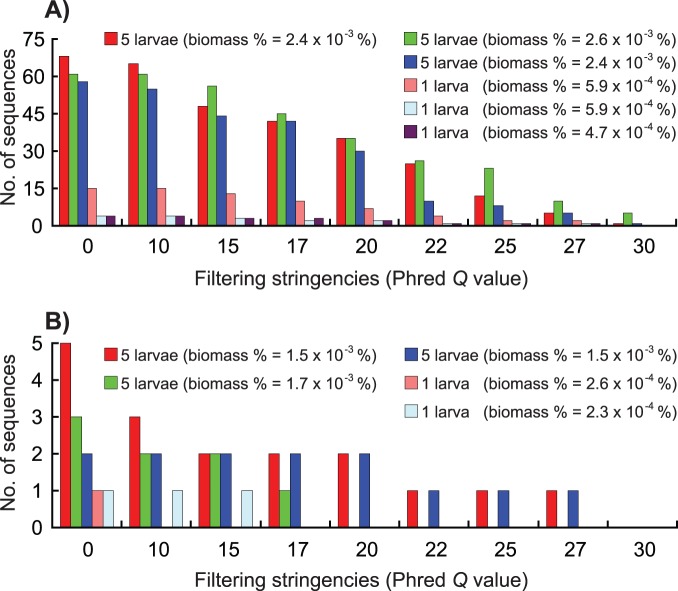
Detection of indicator species spiked into complex communities using a series of filtering stringencies, i.e. *Q* (Phred score)  = 0 (no filtering) to 30. The freshwater species, golden mussel *Limnoperna fortunei*, was spiked into the plankton sample collected from the marine harbour, Bayside (A), while the marine species, bay scallop *Argopecten irradians*, was spiked into the plankton sample collected from the freshwater harbour, Nanticoke (B). For each indicator species, we set up three replicates and four gradients (See Figure 1 for more detail).

For a total of 12 cases (four gradients×three replicates) for each harbour, indicator species were recovered in six and five cases for Bayside (golden mussels spiked) and Nanticoke (bay scallop spiked), respectively (Figure 4). All failed cases involved samples spiked with low quantities of indicator species (i.e. 0.01 and 0.1 larva/sample; Figures 1 & 4), suggesting that the biomass of spiked indicator species was below the detection threshold [11].

Results obtained from the external reference confirmed the findings that rare taxa were eliminated during the sequence filtering process, and such elimination became more severe as filtering stringencies increased (Figure 4). In addition, when examined within each of the two indicator species, these indicator species trended to be eliminated as they became rarer in artificially assembled communities (Figure 4). For example, bay scallops were eliminated at quality score value as low as 10 when the biomass percentage was 2.6×10^−04^%. Similar elimination at low *Q* value (*Q* = 17) was also observed in its replicate (Figure 4). On the contrary, bay scallops were recovered in the cases when higher biomass of indicator species were used (Figure 4). In addition, we found difference between these two indicator species. Even though biomass of these two spiked indicator species was similar in some of the artificially assembled communities, golden mussels were recovered at almost all *Q* values, whereas bay scallops were recovered in a limited number of cases (Figure 4).

## Discussion

High-throughput sequencing technologies are quickly replacing traditional Sanger sequencing as methods for molecular and ecological profiling of complex communities. However, concern has been raised that high-throughput sequencing technologies may introduce artifacts and significantly inflate biodiversity estimates [15]–[20]. Quality filtering is a conventional and convenient method that can remove error-prone sequences [15]–[20]. Results from several studies have indicated that this method could greatly improve biodiversity estimates in environmental samples [16]–[19]. However, as rare species represented by low-abundance sequences may be sensitive to the filtering process, the influence of artifact removal on rare species detection has not been evaluated in natural complex communities. In this study, we employed both internal and external references to evaluate this technical concern. Our results clearly demonstrated that elimination of rare taxa occurred at all filtering stringencies examined, and that more rare taxa were eliminated as filtering stringency increased. Moreover, elimination of rare species intensified as biomass of a species in a community was reduced (Figures 2, 3 & 4).

Differentiation of sequencing errors/artifacts from real sequences in large high-throughput sequencing datasets represents an immense technical challenge, not only because such large datasets require extensive computation but also because we have very limited knowledge on biodiversity in complex communities, and thus lack suitable references for identifying and eliminating errors/artifacts while preserving real sequences [16]–[19]. In this study, we employed both internal and external references to assess effects of artifact removal on rare species detection. Our practice for establishing both internal references from a complex community itself and external references using foreign indicator species provides a practical strategy to calibrate the artifact removal process. In addition, both internal and external references can refer to each other to provide case-specific evaluation since sequencing quality may vary among communities and/or replicates. Such variation was observed between the two communities (Figures 2 & 3) and among replicates (Figure 4) in this study. Based on results obtained from both internal and external references, researchers could estimate that how much “rare biodiversity” was eliminated during data processing, choose parameters based on unique characteristics of each dataset to perform sequence filtering process, and further make corrections for α-diversity estimates for downstream analyses.

Rare taxa may be targets for management, either because they are native taxa of conservation significance or because they may be recently introduced non-indigenous species whose extirpation is deemed desirable. However, detection of rare species represents an enormous technical challenge, especially for multiple species detection in some habitats such as aquatic ecosystems [11 and references therein]. Our earlier study clearly demonstrated that 454 pyrosequencing represents a promising tool for recovery of rare species, as we found that indicator species spiked into plankton communities can be recovered at exceptionally low levels, as low as 2.3×10^−5^% biomass [11]. Using the same assembled communities [11], as well as internal references chosen from complex communities themselves, we found that rare taxa were eliminated during sequence filtering process (Figures 2, 3 & 4). The elimination of rare species intensified as relative biomass of the target species decreased in the assayed community (Figure 4). Rare taxa elimination is easy to overlook, not only because rare taxa are represented by extremely low percentages of sequence reads in extremely large datasets (e.g. Figure 4), but also because quality filtering is usually employed at the beginning of data pre-processing, resulting in the unwitting loss of low-abundance sequences. Meanwhile, unfiltered datasets usually are not processed to serve as references, mainly due to extensive computational demands of these datasets. Our results obtained here suggest that it is crucially important to properly manage high-throughput sequencing data and to use unfiltered datasets as a reference for taxa detection, especially rare species.

Generally, rare species in communities have fewer sequence reads during PCR amplifications than do more common species (e.g. see Figure 4 for biomass gradients and Table 1 in reference 11), although PCR could alter abundance of taxa by biased amplification. Despite that sequencing error ratio may be comparative for both low- and high-abundance taxa, rare taxa represented by low-abundance sequences trend to be discarded first due to the low number of sequences in final datasets. Results obtained in this (Figure 4) and other studies [11], [25] determined that OTUs/taxa represented by low-abundance sequences such as singletons, doubletons and tripletons may be informative and valuable in reflecting rare and/or unique lineages in communities. These sequences may have lower quality (i.e. *Q* values), which may discarded during sequence filtering processes (e.g. Figure 4). Loss of power to detect rare and/or unique lineages in communities could lead to underestimation of biodiversity levels (Fig. 2) and missing targets for management in conservation programs. However, sequences containing sequencing errors are usually believed to appear less abundant [16], [26]. Consequently, technical difficulties still limit accurate sorting of informative low-abundance sequence reads from errors/artifacts. The use of deeper sequencing platforms such as Illumina HiSeq and MiSeq can provide more sequences for some rare taxa, which may potentially solve the problem that we detected in this study. However, deeper sequencing can recover much rarer taxa in complex communities. Consequently, such a technical problem may still exist when using deeper sequencing strategies.

## Conclusion

Our study based on both internal and external references showed three clear patterns: 1) elimination of rare taxa occurred at all filtering stringencies examined, 2) more rare taxa were eliminated as filtering stringencies increased, and 3) elimination of rare species intensified as biomass of a species in a community was reduced. Our study provides a warning that caution should be taken to extract rare taxa from complex communities when using sequence filtering for high-throughput sequencing data. This warning is a call for caution when detecting rare taxa, and for development of powerful mathematical algorithms for data processing. Because the problem detected here may still exist after using deeper sequencing techniques, as well as sequencing quality may vary among communities and replicates, the strategy of setting up both internal and external references here provides a practical way to evaluate the effects of artifact removal on biodiversity measurement. As seen here (Figure 2) and in many other studies [16]–[20], sequencing artifacts can largely inflate biodiversity, artifact removal is still a practical way to get accurate species richness and α-diversity estimates for complex communities so long as researchers are aware of the problem and properly manage results generated for rare taxa in communities.

## Supporting Information

Table S1 **Selected internal Operational Taxonomic Units (OTUs) for the complex plankton communities collected from the marine harbour, Bayside, and the freshwater harbour, Nanticoke.** For all selected internal reference OTUs, the number of sequences in each OTU, the representative sequence of each OTU, and BLAST information including E value, similarity and coverage are shown.
(XLS)

Table S2 **Recovery of internal reference Operational Taxonomic Units (OTUs) for the complex plankton communities collected from the marine harbour, Bayside, and the freshwater harbour, Nanticoke, at a series of filtering stringencies from** ***Q*** **(Phred score)  = 0–30.** Y  =  OTU recovered, N  =  OTU was not recovered.
(XLS)
